# Re-refinement from deposited X-ray data can deliver improved models for most PDB entries

**DOI:** 10.1107/S0907444908037591

**Published:** 2009-01-20

**Authors:** Robbie P. Joosten, Thomas Womack, Gert Vriend, Gérard Bricogne

**Affiliations:** a CMBI, NCMLS, Radboud University Nijmegen Medical Centre, PO Box 9101, 6500 HB Nijmegen, The Netherlands; b Global Phasing, Sheraton House, Castle Park, Cambridge CB3 0AX, England

**Keywords:** re-refinement

## Abstract

The deposition of X-ray data along with the customary structural models defining PDB entries makes it possible to apply large-scale re-refinement protocols to these entries, thus giving users the benefit of improvements in X-ray methods that have occurred since the structure was deposited. Auto­mated gradient refinement is an effective method to achieve this goal, but real-space intervention is most often required in order to adequately address problems detected by structure-validation software. In order to improve the existing protocol, automated re-refinement was combined with structure validation and difference-density peak analysis to produce a catalogue of problems in PDB entries that are amenable to automatic correction. It is shown that re-refinement can be effective in producing improvements, which are often associated with the systematic use of the TLS parameterization of *B* factors, even for relatively new and high-resolution PDB entries, while the accompanying manual or semi-manual map analysis and fitting steps show good prospects for eventual automation. It is proposed that the potential for simultaneous improvements in methods and in re-refinement results be further encouraged by broadening the scope of depositions to include refinement metadata and ultimately primary rather than reduced X-ray data.

## Abstract 2

An evaluation of validation and real-space intervention possibilities for improving existing automated (re-)refinement methods.

## 1. Introduction

The availability of three-dimensional macromolecular co­ordinates is a prerequisite for many types of studies, such as engineering protein function and stability, understanding the molecular origin of genetic disorders, studying intermolecular interactions and designing new drugs, to name only a few. For some of these research fields the accuracy of the coordinates is more important than for others. For example, understanding whether a single-nucleotide polymorphism (SNP) causes an effect that leads to a disease often only requires knowledge of its location in the protein. On the other hand, even small inaccuracies in atomic coordinates can have detrimental effects on predictions of intermolecular contacts in structure-based drug design and on numerous other methods in macromolecular structural bioinformatics that are parameterized on the basis of known protein structures. For example, if the structures that are used to design a docking force field are not very accurate, the force field will not be very accurate and thus docking calculations based on it will be of limited usefulness.

The methods of macromolecular crystallography have im­proved a great deal in recent years (see, for example, Kleywegt & Jones, 2002 ▶). The rapidly growing numbers of increasingly accurate protein structures are aiding methods development in such fields as drug docking (Nabuurs *et al.*, 2007 ▶), molecular dynamics (Hub *et al.*, 2007 ▶) and homology modelling (Krieger *et al.*, 2004 ▶).

It is a truism that every deposited structure has been refined with software that was (at best) state of the art at the time of deposition and that the software itself may not have been used in an optimal manner. It was therefore no great surprise when previous studies in the field of NMR showed that the re-refinement of existing protein structures using methods that have been improved since their deposition can give significantly better structure models than the original ones (Nabuurs *et al.*, 2004 ▶). We have developed a fully automated re-refinement protocol to achieve similar results with structures solved by macromolecular X-ray crystallography (Joosten & Vriend, 2007 ▶). Although this automated re-refinement gives useful results, in this paper we continue to seek further improvements and propose enlarging the scope of what should ideally be deposited.

The validation software used at the Protein Data Bank (Berman *et al.*, 2000 ▶) reports numerous outliers in protein geometry and backbone torsion angles. Other potential problems are recorded by external validation sites such as PDBREPORT (Hooft *et al.*, 1996 ▶) or the Electron Density Server (Kleywegt *et al.*, 2004 ▶). Re-refinement of PDB entries may reduce the number of outliers and improve the fit to the experimental data in terms of *R* and *R*_free_, but large problems cannot be fixed by automated gradient refinement alone. Fortunately, such re-refinement procedures also produce new electron-density and difference-density maps that can be used to manually (and hopefully in the future also automatically) identify and remedy the remaining problems.

In this study, we combined automated re-refinement with validation methods in order to produce a catalogue of problems in PDB entries that are amenable to automatic correction. For this purpose, we have applied the *PDB-REDO* protocol (Joosten & Vriend, 2007 ▶; we had previously performed a re-refinement of 1195 PDB files for which adequate experimental X-ray data to a resolution of 2.00 Å were available using this protocol) to five selected PDB entries followed by manual real-space intervention based on the results of structure-validation routines in *WHAT_CHECK* (Hooft *et al.*, 1996 ▶), *Coot* (Emsley & Cowtan, 2004 ▶) and *pdb-care* (Lütteke & von der Lieth, 2004 ▶). An overview of an independent and complementary activity of monitoring most new PDB depositions since July 2007 for possible model improvements on the basis of electron-density maps and difference maps using the *autoBUSTER* software suite (Bricogne *et al.*, 2008 ▶) is presented in §[Sec sec6]5.

The results of both studies show that there is great potential for the improvement of X-ray structures, especially with the continuously improving software tools that are available. As long as crystallographers keep faithfully depositing their experimental data and critical details of their refinement procedures, the PDB files of X-ray structures can be improved from year to year, thereby strengthening the basis of structural biology and bioinformatics research.

## 2. Methods

### 2.1. Test-case selection

Five PDB entries were selected because they were important in our current homology-modelling projects, have medium- to high-resolution reflection data (ranging from 1.45 to 1.94 Å) that includes an *R*_free_ test set and were recent additions to the PDB. These were 1lf2 (Asojo *et al.*, 2002 ▶), 1zcs (Thapper *et al.*, 2007 ▶), 2ete (Opaleye *et al.*, 2006 ▶), 2qc1 (Dellisanti *et al.*, 2007 ▶) and 2vno (Gregg *et al.*, 2008 ▶).

It must be stated strongly at the outset that these five files were by no means singled out because they were particularly good or bad. They are just examples from a very large group of PDB entries that triggered one of the ‘this can be improved’ diagnostics in one of the validation programs and that we recently needed as templates for in-house projects.

### 2.2. Automated re-refinement

#### 2.2.1. Software

The automated re-refinement procedure uses the *CCP*4 suite (Collaborative Computational Project, Number 4, 1994 ▶), most notably *REFMAC* (Murshudov *et al.*, 1997 ▶), *WHAT_CHECK* and a few dedicated programs (*Cif*2*cif* and *Extractor*, described below). These programs, as well as the re-refinement script, are available at http://www.cmbi.ru.nl/pdb_redo.

#### 2.2.2. Reflection-data preparation

When we re-refined all crystallographic structures in the PDB for which X-ray data to a resolution of exactly 2.00 Å had been deposited, we observed a series of inconsistencies in the reflection-data files from the PDB that made automated use troublesome. Some typical examples were measured reflections reported as amplitudes, intensities or both, missing estimated standard uncertainties, *R*_free_ flags in different formats *etc*.

The program *Cif*2*cif* was written to reformat the reflection data to a consistent format, keeping only the essential information. The *Cif*2*cif* output contains reflection indices (*h*, *k*, *l*), amplitudes (*F*), estimated standard uncertainty values (σ*F*) and the *R*_free_ flag. When necessary, intensities and their sigma values were converted to amplitudes using *F* = *I* ^1/2^ and σ*F* = σ*I*/2*F* (Kleywegt *et al.*, 2004 ▶). If σ*F* values are missing from the input file or when all values present are zero, they are all set to 0.01 in order to avoid technical problems in *REFMAC*.

The program *Extractor* was written to combine relevant information from the experimental reflection-data file and the coordinate file. These data included the reported resolution, *R* and *R*_free_, the resolution range of the data, the unit-cell parameters and the TLS (Schomaker & Trueblood, 1968 ▶) groups used in the original refinement. In cases where TLS was not used in the original refinement or where the TLS groups were not reported, they were defined as one single group per protein chain.

The structure-factor files were converted to MTZ format (a standard used in the *CCP*4 suite) and used to recalculate *R* and *R*_free_ with *REFMAC* using default settings. When needed, ligand-restraint dictionaries were automatically created by *REFMAC*.

#### 2.2.3. Re-refinement

Default *REFMAC* parameters, as specified in the *CCP*4 Graphical User Interface (*CCP*4*i*), were applied with two exceptions: carbohydrate links were only used if they were described in the PDB file and anisotropic *B* factors were refined if ANISOU records were provided. The structure models were first subjected to ten cycles of rigid-body refinement. In our previous study this was needed for a small number of structures that gave large deviations between the recalculated *R*_free_ and the value from the PDB header because of a rotation or translation of the coordinates with respect to the electron density which may follow from a slight mismatch in unit-cell parameters. We retained this non-invasive step to provide a fall-back structure in case further re-refinement was unsuccessful.

TLS refinement was then performed on the rigid-body refined structures. Ten cycles of TLS refinement were per­formed, followed by 20 cycles of restrained refinement in which only the weight of the X-ray terms with respect to the geometric and *B*-factor restraints was changed. Seven different weights were used: 1.00, 0.70, 0.50, 0.30, 0.10, 0.05 and 0.01. This re-refinement resulted in eight models for each structure: one rigid-body refined structure model and seven structure models that were obtained through restrained refinement with TLS.

The best of the seven TLS-refined structure models was selected using the following criteria, in which Δ*R* is used to denote the difference in *R* values (all expressed in units of percentage points or %; see §[Sec sec3.1]3.1): *R*_free_ − *R*.

(i) All models with a root-mean-square deviation from ideal values of bond angles of over 3.0° were rejected.
(ii) Models with Δ*R* greater than 5% were rejected. This rule was relaxed in cases in which Δ*R*_0_, the value of Δ*R* prior to re-refinement, was also greater than 5%. In these cases, the requirement was that Δ*R* did not increase above Δ*R*_0_.
(iii) The model with the lowest *R*_free_ was selected from the remaining candidates. In cases when two or more models had the same *R*_free_ (within 0.1%) that with the smallest Δ*R* was selected.
(iv) In cases where the *R*_free_ of the optimal model was higher than that of the rigid-body refined model (that is, when the structure model actually became worse as a result of re-refinement), all re-refined models were rejected and the rigid-body refined structure model was kept as the final re-refinement result.

#### 2.2.4. Validation and manual real-space intervention

After automated re-refinement had proceeded as described, the resulting structure models were analyzed with *WHAT_CHECK* to search for features that required manual optimization. Special attention was paid to tests for bond lengths, bond angles, missing side-chain atoms, atomic occupancies, alternate side-chain conformations, atomic overlap (bumps), residue packing and hydrogen bonding. These tests were based on the atomic parameters only and can therefore be used on any structure model with or without experimental data.

*Coot* was used to evaluate anomalies detected by *WHAT_CHECK* in the context of the experimental data. The ‘Check/Delete Waters’ routine and peaks in the *mF*_o_ − *DF*_c_ difference electron-density maps were used to detect additional problem areas in the re-refinement results. When necessary, these anomalies were resolved by side-chain refitting and the addition or removal of waters, alternate side-chain conformations or hetero compounds. The resulting structure models were refined in *REFMAC* using five cycles of TLS refinement followed by five cycles of restrained refinement with default settings for *REFMAC* in *CCP*4*i* and with the optimal geometric restraint weight found during the previous automated re-refinement stage. This cycle of rebuilding and refinement was performed three times. The final structures were validated with *WHAT_CHECK* and *pdb-care*.

## 3. Results

### 3.1. Overall re-refinement results

The process of automated re-refinement followed by manual rebuilding resulted in five structure models with similar or improved *R*_free_ values. Table 1 ▶ shows the validation scores for the original, re-refined and manually optimized structures.

In order to avoid all misunderstandings in the forthcoming comparisons of *R* values, which are traditionally expressed in terms of percentage points (%), we wish to state explicitly that the changes mentioned in the text are absolute changes in percentage point (%) units rather than relative changes as a percentage of a reference value. For instance, a 5% improvement to an *R* value of 20% would produce *R* = 15% (*i.e.* a decrease of five percentage points from the initial value) and not *R* = 19% (*i.e.* a decrease of 5% of the initial value). The same applies to the outline of the re-refinement logic presented in §[Sec sec2.2.3]2.2.3.

### 3.2. PDB entry 1lf2

The automated re-refinement of PDB entry 1lf2 resulted in an *R*_free_ improvement of 5.6% and an improvement of the Ramachandran and rotamer normality scores. However, the side chains were distorted, with planarity deviations that were much larger than the standard uncertainties in the *WHAT_CHECK* geometry library. 14 H atoms (all glutamine H^∊^ atoms with a *B* factor of exactly 20.00 Å^2^) were observed in the original PDB entry. The arbitrary nature of the presence of only these 14 protons and the fact that there is no mention of these protons in the associated article led us to believe that they were most likely a remnant of an experiment during the refinement procedure. We therefore believe that the automatic removal of these protons is not a problem.

*WHAT_CHECK* detected a range of atoms in residues 237*A*–244*A* with occupancies of 0.50 but without alternate positions. The difference map showed positive density of up to 6.0σ at these atoms. Resetting the atomic occupancies to 1.00 removed nearly all these difference density peaks.

Evaluation of the hydrogen-bond network showed 28 waters without hydrogen bonds; these 28 waters were removed. Additional validation of waters in *Coot* resulted in 56 further waters being removed during manual optimization of the structure.

The second and fifth highest peaks in the difference map, one on a twofold-symmetry axis and the other near Lys238*A*, were nearly tetrahedral. They were assumed to be sulfates because the original publication of the 1lf2 structure mentions the use of ammonium sulfate in the crystallization medium.

**Figure 1:**
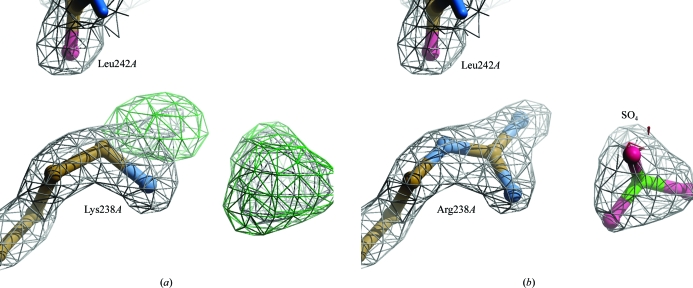
Detail of PDB entry 1lf2 after one round of manual optimization (*a*) and the final model (*b*) in 2*mF*_o_ − *DF*_c_ electron-density maps (contoured at 2.0σ) and *mF*_o_ − *DF*_c_ difference density (contoured at 4.0σ). Based on the crystallization conditions described in Asojo *et al.* (2002 ▶), the tetrahedral difference-map peak in (*a*) was modelled as a sulfate. Lys238*A* was modelled as an arginine based on the (difference) density map contours. The H^∊^ of this arginine in (*b*) makes a hydrogen bond to the backbone carbonyl of Leu242*A*. The figures were created with *Coot* (Emsley & Cowtan, 2004 ▶) and *Raster*3*D* (Merritt & Bacon, 2007 ▶).

After one cycle of manual optimization and refinement the third largest difference-map peak was located near the side chain of Lys238*A*, indicating a post-translation modification or a possible sequence error. Modelling Lys238*A* as an arginine gave an improved fit to the electron-density map and removed the difference-map peak (Fig. 1 ▶).

The final refinement resulted in an *R*_free_ improvement of 6.5% compared with the original PDB entry. All *WHAT_CHECK* quality scores were also improved, most notably the side-chain rotamer normality score and the side-chain planarity.

### 3.3. PDB entry 1zcs

The automated re-refinement of 1zcs resulted in a small improvement of *R*_free_ by 0.3%. Geometric quality scores also did not show large changes.

**Figure 2:**
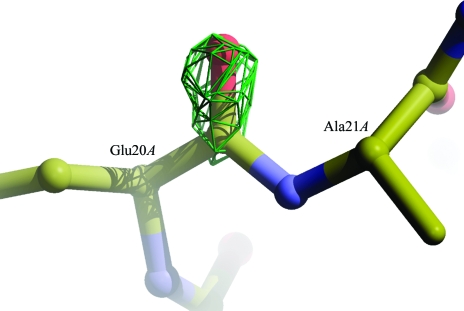
Detail of PDB entry 1zcs after automated re-refinement. The carbonyl atoms of Glu20*A* were modelled with 50% occupancy but without any alternate atom positions by Thapper *et al.* (2007 ▶). This results in a positive *mF*_o_ − *DF*_c_ difference-map peak on this carbonyl group, contoured here at 3.0σ. The difference-map peak disappeared after setting the atomic occupancies to 1.00.

*WHAT_CHECK* reported 52 residues that had atoms with partial occupancies. Most of these were side-chain atoms with occupancies of 0.50 and poor electron density. No alternate conformers were supplied for these side chains. The carbonyl atoms of residue 20*A* also had occupancies of 0.50, whereas a positive difference-map peak near these atoms suggested that the site was fully occupied (Fig. 2 ▶). All these occupancies were reset to 1.00.

The difference map showed large positive peaks on the Fe atoms of two iron–sulfur clusters. Owing to a software problem in the re-refinement, they had been refined as N atoms. These peaks disappeared after subsequent refinement with a corrected version of *REFMAC*. TLS groups were added for the (molyb­dopterin-cytosine dinucleotide-*S*,*S*)-dioxo-aqua-molybdenum(V) ligand and the two iron–sulfur clusters.

The final refinement resulted in an improvement in *R*_free_ of 2.5%. Overall, re-refinement caused little change in the *WHAT_CHECK* quality scores apart from a small increase in the number of atomic overlaps and a slight improvement in the rotamer normality.

### 3.4. PDB entry 2ete

Prior to any re-refinement, the calculated *R*_free_ was found to be 1.1% lower than the value from the PDB header, whereas *R* was nearly equal to the value from the header. Additionally, the calculated values of *R* and *R*_free_ differed by only 0.1%. This indicated that there was something wrong with the *R*_free_ set specified in the experimental data file. As a result, the calculated *R* and *R*_free_ could not be used as reference values to assess the result of the re-refinement. The values extracted from the PDB header were used instead.

The automated re-refinement of 2ete resulted in a 1.5% improvement of *R*_free_ at the expense of the geometric quality of the structure. The bond lengths and angles deviated much more from ideal values than they did before the re-refinement. The side-chain planarity r.m.s.*Z* score of 1.04 was poor.

Closer inspection of the *WHAT_CHECK* validation report showed that many geometric outliers could be traced to residues *B*94 and *B*166, two threonines with inverted C^β^ chirality. Inspection of the electron-density maps showed that both side chains were poorly fitted in the original PDB entry, with χ_1_ torsion angles that deviated by 180° from the optimal values. They had been forced into their awkward conformation by the automated refinement. Apart from these outliers, a cell-scaling problem was diagnosed as the possible reason why the bond lengths were systematically slightly longer than ideal.

**Figure 3:**
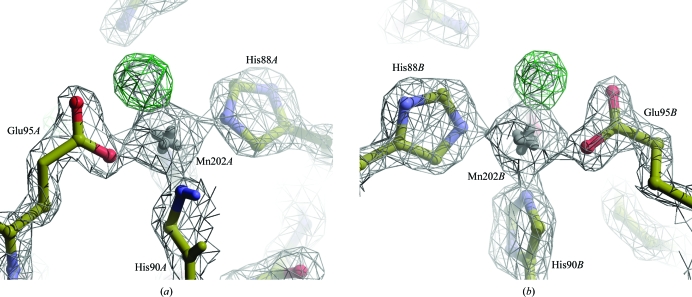
Mn atoms in PDB entry 2ete after automated re-refinement in 2*mF*_o_ − *DF*_c_ electron-density maps (contoured at 2.5σ). Both chain *A* and chain *B* show a clear positive difference-density peak (contoured at 5.0σ) at a manganese-coordination site. Placing waters at these peaks removed the difference density.

The difference map showed a large number of peaks, six of which were over 10.0σ. The two largest peaks in the difference map were interpreted as waters coordinating two manganese ions (Fig. 3 ▶). Other large difference-map peaks were caused by poorly fitted side chains and by a large number of unassigned water peaks. 70 waters could be unambiguously assigned on the basis of these peaks.

The final refinement round resulted in an *R*_free_ improvement of 2.8%. The side-chain rotamer score increased, but the backbone and Ramachandran scores decreased. The geo­metric deviations from ideality were still larger than in the original PDB entry, but as all r.m.s.*Z* scores were well below 1.00 we saw no need to use tighter geometric restraints.

### 3.5. PDB entry 2qc1

The automated re-refinement of 2qc1 resulted in an *R*_free_ improvement of 0.9%. The validation report showed small improvements in the quality *Z* scores except for the side-chain rotamers. The side-chain planarity was distorted.

**Figure 4:**
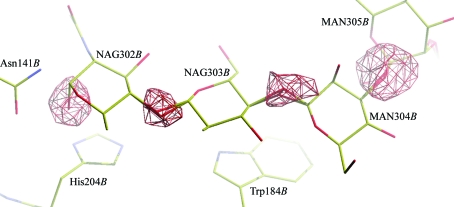
The first four carbohydrates of the N-linked glycan in PDB entry 2qc1 with four negative *mF*_o_ − *DF*_c_ difference density peaks at their O1 atoms. The difference density map is contoured at −3.0σ and waters are not shown for clarity. The O1 atom in NAG302*B* was placed in an axial conformation in the original PDB entry, but should have been removed completely before deposition. This superfluous oxygen hampers the detection of the link to Asn141*B* in *Coot*. The other O1 atoms are were left on top of other O atoms when creating links between the carbohydrates, which resulted in difference density peaks and numerous atomic overlap warnings in *WHAT_CHECK* (Hooft *et al.*, 1996 ▶). Additionally, MAN304*B* is a β-d-mannose and must be named BMA instead of MAN.

The most striking validation result was the large number of problematic atomic overlaps, the worst of which was a 2.21 Å overlap between two mannose O atoms that were part of a large N-linked glycan. A development version of *pdb-care* showed many more problems in this carbohydrate structure. The O atoms that are eliminated when a carbohydrate link is formed had been left in the original PDB entry. Ten such superfluous O atoms were detected, each of which was associated with a strong negative difference-map peak (Fig. 4 ▶). Furthermore, residue *B*304, a β-d-mannose, was named MAN instead of BMA.

Apart from removing the superfluous O atoms and refitting one poorly fitted tryptophan (residue *B*176), little real-space intervention was needed. The final *R*_free_ was 1.4% lower than before re-refinement. Most quality scores improved and the number of atomic overlaps was reduced by 40%.

### 3.6. PDB entry 2vno

The *R* and *R*_free_ values from the PDB header (18.0% and 22.6%) could not be reproduced, the recalculated values coming out at 19.5% and 24.1%, respectively. Automated re-refinement did not improve these values. The first manual optimization cycle was therefore based on the rigid-body refined structure.

*WHAT_CHECK* validation of this structure showed some large geometry outliers which resulted in a bond-length r.m.s.*Z* of 1.40 and a side-chain planarity r.m.s.*Z* of 2.01. A number of residues also had missing atoms in the middle of side chains. For instance, residue *A*176 was a glutamate with missing C^γ^ and C^δ^ atoms, even though the O^∊^ atoms were present.

**Figure 5:**
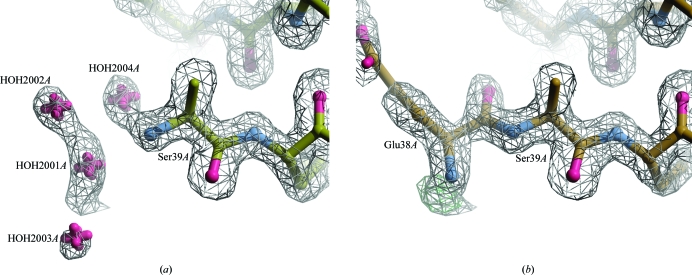
N-terminus of chain *A* in PDB entry 2vno after automated re-refinement (*a*) and manual optimization (*b*). The waters near the N-terminus of chain *A* occupied electron density that was occupied by a glutamate in the NCS-related chain *B*; the electron density (contoured at 1.0σ) was not fully connected. The waters were removed and replaced by an N-terminal glutamate. After further refinement, connected electron density was obtained for the glutamate backbone.

Analysis of waters using *Coot* showed a number of questionable waters near the N-terminus of chain *A*. In the non­crystallographic symmetry-related chain *B* these positions were occupied by a glutamate residue. The waters near chain *A* were replaced by a glutamate residue (Fig. 5 ▶). A total of 42 waters were removed.

Refinement of the anisotropic *B* factors in combination with the TLS models led to over-parameterization and subsequent refinement steps were only successful after the ANISOU records had been removed. The final *R*_free_ was 0.6% lower than the value from the PDB header and all quality indicators had improved. Based on validation with *pdb-care*, residues 1210*A* and 1211*B* were renamed from GAL to GLA in accordance with the remediated PDB format (Henrick *et al.*, 2008 ▶).

## 4. Discussion

**Figure 6:**
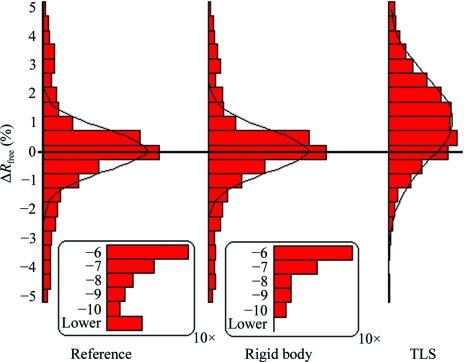
Distributions of Δ*R*_free_ for 1195 X-ray structures from the PDB with a reported resolution of 2.00 Å. Δ*R*_free_ = *R*_free, org_ − *R*_free, recalc_, where *R*_free, org_ values were taken from the PDB-file headers and *R*_free, recalc_ values were recalculated after subsequent refinement steps: (i) simple recalculation of *R*_free_ without any additional refinement, (ii) rigid-body refinement to compensate for any accidental translation or rotation of the coordinates before deposition and (iii) refinement with TLS models for atomic displacement. TLS models in PDB headers were kept if available; in all other cases one group per chain was used. The Gaussian fits through these histograms are for visual reference only. The average *R*_free_ values reported in the PDB are 24.2%. We recalculated these values without additional refinement and obtained an average of 24.5%. After rigid-body refinement and TLS refinement, the average *R*_free_ values were 24.3% and 23.0%, respectively.

In a previous experiment (Joosten & Vriend, 2007 ▶), we had shown that a simple automated re-refinement protocol could improve the *R*_free_ values of 78% of 1195 PDB entries (all with 2.00 Å resolution) for which the X-ray reflection data and the *R*_free_ set could be reconstructed from the deposited data (Fig. 6 ▶). It is obviously our goal to obtain the best possible atomic coordinates from the available data. We therefore decided to manually optimize five structure models in order to see how the refinement affected the electron-density maps and to search for improvements that are simple enough to be automated in the next re-refinement round.

We encountered several problems in the automated and manual re-refinement, the most important being the lack of information about the original refinement protocol. This causes problems when trying to reproduce the refinement results, as shown with the calculated *R*_free_ value of 2ete. Because the calculated *R*_free_ deviated from the PDB-header values but the calculated *R* did not, it is likely that the wrong set of reflections was flagged as the *R*_free_ set. This has implications for the re-refinement results: because the *R*_free_ reflections have been used in the model-building process, the *R*_free_ values after re-refinement are biased. The general lack of sufficient ‘metadata’ about the original refinement makes it difficult to set up regular large-scale re-refinements for new PDB entries using the given versions of refinement and rebuilding software or, conversely, large-scale benchmarks for new versions of the software against the same set of entries.

The differences between the calculated *R* and *R*_free_ and the values from the PDB header are small for the five PDB entries discussed here. In the case of 1zcs, a small deviation was caused by applying the wrong solvent model. Applying the bulk-solvent model of *REFMAC* removed this deviation. For 1zcs, the solvent model should have been recognized by the re-refinement protocol because it is described in the PDB header. This error has been corrected in our scripts. In contrast, no information about the solvent model was reported for 2qc1. Therefore, other sources of *R*-factor deviations should be considered. Kleywegt *et al.* (2004 ▶) have discussed many possible causes of such discrepancies. Here, typical issues are the resolution and *F*/σ*F* cutoffs that were applied when selecting the reflections used in the original refinement and subsequent calculations of *R* and *R*_free_. As the *PDB-REDO* protocol always uses all available reflections, slightly different sets of reflections may be used in the original *versus* our current calculation of *R* and *R*_free_.

Much larger differences between calculated *R* values and values from the PDB header occur than were found in this study. Such deviations do not necessarily mean that the atomic coordinates are poor, but may rather indicate that there is something wrong with the deposited reflection data. One such instance, PDB entry 3d0b (Barta *et al.*, 2008 ▶), will be mentioned below in §[Sec sec5]5.

Hetero compounds are troublesome entities for re-refinement and structure validation. Their geometric restraints are not deposited in the PDB, which means that refinement programs have to generate restraints for all compounds not described in their restraints library. This typically requires the interpretation of atomic coordinates in a PDB file to assign bond types, protonation states and possible charges and to detect other features such as aromatic ring systems. This can lead to different geometric restraints being used by different refinement programs and thus to different (re-)refinement results. Validation software suffers from the same problems. These issues may be addressed by creating a standard re­pository of geometric restraints for hetero compounds, similar to those of Engh & Huber (1991 ▶) for proteins and of others for nucleic acids (Parkinson *et al.*, 1996 ▶). However, new compounds are added to the PDB every week, which makes it problematic to keep such a repository up to date.

Carbohydrates have their own problems, some of which have recently been resolved in the PDB remediation. Compounds that only differ in the chirality of one atom now have unique names. For instance, α-d-mannose (MAN) and β-­d-mannose (BMA) only differ in the chirality of the C1 atom. Before the remediation, the single hetero-compound name MAN was used for both compounds, even though the compound BMA was described in the PDB ligand dictionary. Unfortunately, compounds that were previously refined with the wrong chirality have now been renamed to make the compound name match the coordinates, thereby perhaps casting in stone a certain number of errors. We are working on a *pdb-care*-based method that can detect such problems and give suggestions for (automated) correction. After updating the compound name, the coordinates can be refined with the correct chirality.

A number of the real-space interventions applied to the five evaluated PDB entries can be automated. In fact, several tools already exist for side-chain refitting (DePristo *et al.*, 2005 ▶) and density fitting of water molecules (Perrakis *et al.*, 1997 ▶). However, these tools should be applied with great care. For example, some of the ‘free atoms’ placed into density during the automatic model building with *ARP*/*wARP* (Perrakis *et al.*, 1999 ▶) of PDB entry 2vno were placed at positions that should have been assigned to protein side-chain atoms; however, the automated interpretation process failed to identify them as such and they were left as ‘waters’ while distributed differently from true waters. On the other hand, too few waters were fitted in the case of 2ete. These examples show that a careful re-evaluation of waters in structure models is necessary in the automated re-refinement protocol. Implementing this is not trivial; automated water fitting requires sensitive parameter optimization and thorough validation of the putative waters.

The two missing waters that appeared at manganese co­ordination sites in 2ete revealed another difficulty: waters in ion-coordination sites behave differently from other waters, with their *B* factors being markedly lower and their coordination distances being much shorter than regular hydrogen-bonding distances. This can erroneously trigger distance cutoffs in water-fitting routines and bump-detection routines in validation software.

Less invasive real-space interventions, such as small geometry fixes, occupancy fixes and side-chain flips to optimize the hydrogen-bonding network, can be applied automatically with little risk of introducing new problems. Of course, these will have little effect on quality estimators such as *R* and *R*_free,_ but that does not mean that they are unimportant.

Filling positive difference-map peaks with compounds other than water, as shown here in 1lf2, requires knowledge of the crystallization conditions and of the cryoprotectants used. This information can be stored in ‘REMARK 280’ records in the PDB header, as was performed for three of the five PDB entries in this study. Even though this record is not ideal for automatic interpretation, it may prove invaluable in future automated re-refinement efforts and the PDB might consider designing an appropriate ontology for these REMARKs.

The mutation in plasmepsin-2 in 1lf2 is an example of a real-space intervention that cannot be automated (yet). Sequence retrieval in UniProt (Leinonen *et al.*, 2004 ▶) showed only distant homologues of plasmepsin-2 with an arginine instead of a lysine residue at position 238 in the sequence. However, this change can be caused by a point mutation in the plasmepsin-2 expression system. To safely fix this possible sequence error, help from the original depositor is required to verify the proposed sequence. It is therefore important that issues such as this be recognized by validation software before deposition of the structure model. Of course, this is not limited to possible sequence errors; structure validation before deposition is always more effective than after deposition. For example, shortly before *WHAT_CHECK* became available to automatically flag space-group-related problems, Kleywegt *et al.* (1996 ▶) discovered a space-group problem in PDB entry 1chr (Hoier *et al.*, 1994 ▶). The problem was corrected and the improved structure was submitted as 2chr. The correction of this type of error is still beyond the scope of automation. Had *WHAT_CHECK* been available in 1994 and had it been used by the original crystallographers, the present situation of having the incorrect 1chr in the PDB database next to the much better 2chr could have been avoided.

## 5. An alternative approach to detecting problem regions in deposited models

In parallel to the investigations reported above on deposited structures that were flagged by structure-validation programs, since July 2007 one of the authors (TW) has been carrying out a regular process of refining most new PDB depositions on a weekly basis, as they are received, using the *autoBUSTER* software (Bricogne *et al.*, 2008 ▶). The selection of structures for re-examination is made by analysing the post-refinement difference map rather than the geometry of the deposited model; this shows up different kinds of modelling issues. The five PDB entries examined above were also analyzed with *autoBUSTER*, with essentially identical conclusions, although the final *R* values achieved were adversely affected by the fact that *autoBUSTER* does not yet allow TLS refinement. The two entries with NCS (2ete and 2vno) were refined with the *autoNCS* option based on local structure similarity restraints (LSSR), as described in Smart *et al.* (2008 ▶).

Looking at the structures with the highest peaks in their difference maps tends to select high-resolution structures containing unmodelled ions, which can often be recognized by considering their observed coordination and the crystallization conditions. These may be deemed biologically irrelevant, but in 2qae (C. Werner, R. L. Krauth-Siegel, M.T. Stubbs & G. Klebe, unpublished work) an unmodelled sodium appears to mediate a contact between the main chain and an FAD and there is a loop in 3czk (Kim *et al.*, 2008 ▶) that has been built into density where a caesium ion clearly ought to be. It also shows up a plethora of unmodelled waters. Even high-resolution structures often show large numbers of unambiguous and even NCS-conserved unmodelled waters; often, such a peak owing to water indicates the correct orientation of a nearby histidine through the implied hydrogen bonding.

Dipoles of difference density along the axis of the C=O bond, which can be detected automatically, are a good diagnostic for flipped peptides. Looking for difference-map peaks in the vicinity of ligands shows up both incorrectly modelled buffer molecules [2q5b (Y. S. Bukhman-DeRuyter, R. Fromme, I. Grotjohann, B. Schlarb-Ridley, H. Mi & P. Fromme, unpublished work), at 1.45 Å resolution, has a glycerol modelled as an acetate] and the occasional situation where the ligand is not as claimed. The deposited structure factors for 3d0b appear to be from a crystal in which a ligand significantly different from that modelled is bound. The reported *R* is 20.0%, whereas we calculated an *R* of 28.7%. Review of the electron-density maps of 3d0b showed an unmistakable 3,4,5-trimethoxybenzyl group which was not part of the chemical structure of the purported ligand, indicating that the reflection data belonged to the correct protein but crystallized with a different ligand than that described in the coordinate file. This is just one example of the problems that can be encountered with liganded protein structures. A recent review by Davis *et al.* (2008 ▶) discusses more examples.

For lower resolution structures, more substantial interpretation errors or insufficiencies are often pointed out by this process, such as register errors, unmodelled density at the termini or extra copies of ligands. This independent monitoring of incoming PDB depositions supports the conclusion that there is a strong case for extending the scope of depositions so that they specify all the relevant information required to reproduce the refinement steps that led to the deposited results and to subsequently repeat them with later versions of the software that may produce improved results from the deposited X-ray data. Such a database of reproducible refinements would be a great asset to software developers, in that it would simplify the large-scale benchmarking of pro­gress in refinement algorithms. In small-molecule crystallo­graphy it has become common practice to no longer even look at electron density and only a few problematic structures still require the attention of experienced crystallo­graphers. We can imagine that our results might provide a small step towards achieving the same situation in the future of macromolecular crystallo­graphy.

## 6. Outlook

This proposal of extended depositions naturally leads to the consideration of X-ray data themselves. In the same way that deposited coordinates are only the best results that could be obtained from the deposited X-ray data by the refinement protocols available at the time and are therefore improvable as these protocols become more sophisticated, those deposited X-ray data are only the best summary of sets of diffraction images according to the data-reduction programs and practices available at the time they were processed.

Just like refinement software, those programs and practices are subject to continuing developments and improvements, especially in view of the current interest and efforts towards better understanding radiation damage during data collection and in taking it into account in the subsequent processing steps. It is therefore natural that the deposition of X-ray data should go beyond its present form and endeavor to collect the diffraction images themselves, together with sufficient information to enable any investigator to retrace the steps of the entire structure-determination process. The Joint Center for Structural Genomics archive (http://www.jcsg.org) is an excellent prototype of what can be achieved in this respect, and its often-acknowledged value to software developers is a clear indication of the potential benefits of such an extended deposition scheme through the dual improvements it would enable in both the results for structures already solved and in the ability to solve new more difficult ones in the future thanks to better tested and better validated software advances.

## 7. Conclusion

Ongoing improvements in crystallographic software and validation tools, combined with the deposition of X-ray data into the PDB, have enabled the development of automated re-refinement protocols, such as that described here, which can improve most structure models compared with their initially deposited form. We have shown examples of real-space interventions that must be incorporated into this protocol to increase its effectiveness.

The rate at which greater sophistication can be achieved in these re-refinement and validation methods will depend greatly on the success of eliciting more information, or ‘metadata’, from the depositors about the protocols they followed: ultimately, from the raw X-ray data to their refinement results. We are aware that this will make the deposition process more time-consuming; however, users of the PDB and software developers will greatly benefit from this extra effort, as it will turn what was previously a static archive of frozen models into a repository of self-improving results through the steady progress in methods developments it will catalyse. Depositors will also benefit from such a paradigm shift, because it will make their structural results more ‘future-proof’, leading to more citations and to higher visibility of their work.

## Figures and Tables

**Table 1:** Validation scores for original, re-refined and manually optimized structure models

PDB code	1lf2	1zcs	2ete	2qc1	2vno
Resolution (Å)	1.80	1.45	1.75	1.94	1.45
*R* (%)					
Original†	24.0 (19.5)	18.0 (15.8)	18.2 (18.1)	22.7 (21.4)	19.5 (18.0)
Re-refined	17.6	17.3	15.9	19.2	19.5
Manually optimized	17.4	15.2	14.8	18.6	18.7
*R*_free_ (%)					
Original†	27.8 (25.8)	19.8 (18.0)	18.3 (19.4)	24.8 (23.3)	24.1 (22.6)
Re-refined	22.2	19.5	17.9	23.9	24.1
Manually optimized	21.3	17.3	16.6	23.4	22.0
Non-water atoms					
Original	2658	6955	3020	2426	2753
Re-refined	2644	6955	3020	2426	2753
Manually optimized	2663	6981	3057	2434	2781
Waters					
Original	338	1002	308	187	574
Re-refined	338	1002	308	187	574
Manually optimized	254	1005	378	193	532
Atomic overlaps‡					
Original	59	72	23	145	58
Re-refined	64	72	31	143	58
Manually optimized	53	87	23	87	42
Packing quality§					
Original	−1.94	−1.51	−1.97	−1.87	−2.38
Re-refined	−1.96	−1.53	−1.98	−1.79	−2.38
Manually optimized	−1.83	−1.50	−1.97	−1.79	−2.30
Ramachandran§					
Original	−1.86	0.07	0.05	−0.62	0.24
Re-refined	−1.43	0.05	−0.23	−0.26	0.24
Manually optimized	−1.44	0.06	−0.16	−0.17	0.39
Rotamer normality§					
Original	−2.38	0.51	0.26	−0.26	−0.11
Re-refined	−1.83	0.42	0.28	−0.37	−0.11
Manually optimized	−1.24	0.63	0.67	−0.39	0.28
Backbone conformation§					
Original	−3.87	−1.90	−2.31	−1.44	−2.18
Re-refined	−3.61	−1.95	−2.56	−1.31	−2.18
Manually optimized	−3.37	−1.82	−2.57	−1.29	−2.04
Bond r.m.s.*Z*					
Original	0.74	0.46	0.43	0.38	1.40
Re-refined	0.96	0.49	0.95	0.96	1.40
Manually optimized	0.61	0.40	0.80	0.70	0.60
Angle r.m.s.*Z*					
Original	0.94	0.69	0.60	0.75	0.87
Re-refined	1.00	0.68	0.83	0.93	0.87
Manually optimized	0.80	0.65	0.76	0.81	0.87
Side-chain planarity r.m.s.*Z*					
Original	0.83	0.59	0.38	0.26	2.01
Re-refined	1.23	0.66	1.04	1.15	2.01
Manually optimized	0.71	0.55	0.86	0.80	0.90

† Calculated from experimental data; PDB header values are given in parentheses.

‡ van der Waals overlap greater than 0.40 Å.

§ *WHAT_CHECK Z* scores.
